# Chance long-distance or human-mediated dispersal? How *Acacia s.l. farnesiana* attained its pan-tropical distribution

**DOI:** 10.1098/rsos.170105

**Published:** 2017-04-12

**Authors:** Karen L. Bell, Haripriya Rangan, Manuel M. Fernandes, Christian A. Kull, Daniel J. Murphy

**Affiliations:** 1 Royal Botanic Gardens Victoria, Melbourne, Victoria 3004, Australia; 2 Australia India Institute and School of Geography, University of Melbourne, Victoria 3053, Australia; 3 Monash Indigenous Centre, Monash University, Victoria 3800, Australia; 4 Centro de Estudos de Geografia e Ordenamento Território, Universidade do Porto, Portugal; 5 Institut de Géographie et Durabilité, Université de Lausanne, Lausanne 1015, Switzerland

**Keywords:** *Acacia farnesiana*, cryptogenic species, human-mediated dispersal, pan-tropical species, transoceanic dispersal, *Vachellia farnesiana*

## Abstract

*Acacia s.l. farnesiana*, which originates from Mesoamerica, is the most widely distributed *Acacia s.l.* species across the tropics. It is assumed that the plant was transferred across the Atlantic to southern Europe by Spanish explorers, and then spread across the Old World tropics through a combination of chance long-distance and human-mediated dispersal. Our study uses genetic analysis and information from historical sources to test the relative roles of chance and human-mediated dispersal in its distribution. The results confirm the Mesoamerican origins of the plant and show three patterns of human-mediated dispersal. Samples from Spain showed greater genetic diversity than those from other Old World tropics, suggesting more instances of transatlantic introductions from the Americas to that country than to other parts of Africa and Asia. Individuals from the Philippines matched a population from South Central Mexico and were likely to have been direct, trans-Pacific introductions. Australian samples were genetically unique, indicating that the arrival of the species in the continent was independent of these European colonial activities. This suggests the possibility of pre-European human-mediated dispersal across the Pacific Ocean. These significant findings raise new questions for biogeographic studies that assume chance or transoceanic dispersal for disjunct plant distributions.

## 1. Introduction

*Acacia s.l.* is a pan-tropical genus with around 1450 species distributed across Africa, Asia, the Americas and Australia [1]. Some of these acacia species are hypothesized to have been transported between continents through long-distance oceanic dispersal [2,3], but in most cases, humans have been the main agents of plant movement in *Acacia* (e.g. [2,4–6]).

This study investigates the relative roles of chance long-distance and human-mediated dispersal in the pan-tropical distribution of *Acacia s.l. farnesiana* (L.) Willd. It is identified as the most widely distributed of all *Acacia s.l.* species in the world [7], and is found in the Americas, the Caribbean, southern Europe, Africa, islands in the Atlantic, Indian and Pacific Oceans, West, South and Southeast Asia, and Australia [6,8]. According to Richardson & Rejmánek [9], *A. farnesiana* is currently regarded as invasive in 12 out of 15 geographical regions globally. Its seeds have been shown to maintain viability after more than a century [10]. Although the new nomenclature of *Acacia* classifies *A. farnesiana* in the genus *Vachellia*, in this study we use the name *Acacia farnesiana* to maintain consistency with historical references that compared it with the original type species known from Egypt (*Acacia nilotica*).

The biogeographic literature identifies the native range of *A. farnesiana* in the Americas, extending from Brazil and Peru to Mexico and the semi-arid regions of southern USA. Its status in the Caribbean is cryptic, and it is considered alien in Argentina [11–13]. This literature, however, does not offer much information about when and how *A. farnesiana* may have dispersed from the Americas and attained its current pan-tropical distribution.

In this study, we used genetic analysis and historical accounts to investigate the dispersal of *A. farnesiana* from the Americas to other parts of the world. We hypothesized that: (i) the introductions to southern Europe were via colonial interactions with the Americas and (ii) introductions to Africa, South Asia, Southeast Asia and Fiji were from southern European sources. In the case of Australia, we tested three alternative hypotheses to explain the pre-British presence of the species in the continent: (i) arrival via Southeast Asia through colonial Portuguese or Spanish interactions; (ii) direct arrival from the Americas through European voyagers or colonial interactions or (iii) pre-European arrival either through oceanic or human-assisted dispersal.

### 1.1. Acacias in human history

While recent movements of *Acacia s.l.* have been for utilitarian purposes like land stabilization [9], acacias have a deep historical association with humans. The species are recorded as being used for building, medicinal and cultural purposes in ancient Africa, the Middle East and the Indian subcontinent [14]. *Acacia nilotica* (L.) Delile was depicted in tomb paintings of Ancient Egypt of the second millennium BCE [15–17], identified in ancient medical treatises of the Indian subcontinent [18], and referred to in the Bible as *shittim* [19]. Early Greek treatises such as the *Enquiry into Plants* by Theophrastus (*ca* 350 BCE) and *De Materia Medica* by Dioscorides (50–70 CE) referred to the tree as *akakía*, a name alluding to its characteristic spiky thorns, and mentioned it as growing in Egypt [20,21].

When early Spanish and Portuguese explorers in the Americas encountered plants that looked similar to previously described and well-known Old World species such as *Acacia nilotica*, they also classified the New World species as acacias [22,23]. One of the first detailed descriptions of an acacia from the Americas was in 1625 by Tobias Aldini. He included an illustration and description of a plant growing in Cardinal Odoardo Farnese's garden in Rome which he named *Acacia Indica Farnesiana*, followed by an illustration and description of *Acacia aegyptiae* (*Acacia nilotica*) to show the features that distinguished the two from each other. He noted that the seeds of this tree had been obtained from the island of Santo Domingo and germinated in 1611 in Farnese's garden [24]. Subsequent botanical texts followed Aldini's presentation of *Acacia farnesiana* alongside *Acacia nilotica* for purposes of comparison [25,26]. The physical similarities to the Old World species, combined with other aesthetic features such as the delicate fragrance of its flowers, may have provided the impetus for the introduction of *Acacia farnesiana* into Europe.

*Acacia farnesiana* is a thorny shrub, between 2 and 3 m in height. Its yellow, ball-like inflorescences bear a delicate scent which is highly valued in the perfume industry [27]. The species has diverse common names around the world, such as sweet acacia, *huisache* or *aromo* in North America, mimosa bush in Australia or *cassier* in France [28]. Its flowers are used in the essential oil industry, with Egypt currently being the largest producer [29,30]. The plant has a range of uses in traditional medicines, tanning, and its leaves and pods are considered as good forage for cattle and ungulates [31].

Despite the existence of various historical texts that provide comparisons between *A. farnesiana* and *A. nilotica* and indicate the role of human-assisted dispersal, biogeographic studies generally feature chance dispersal and other ecological factors for species with pan-tropical distributions. This is particularly so for species such as *A. farnesiana* that have dispersed to Australia. Although early British explorers identified the plant as already present in the continent prior to European colonization, they offered no speculation or explanation of how it may have arrived from the Americas [4,32]. Subsequent biogeographic studies of *A. farnesiana* in Australia have typically attributed its pre-British presence in the continent to transoceanic dispersal and not considered the possibility of precolonial human-assisted dispersal from the Americas. However, there is growing linguistic and genetic evidence for some plant species being introduced from the Americas into Oceania by Austronesian sailors in pre-Columbian times (e.g. [33,34]). There is also increasing evidence of ancient Aboriginal influence on the biogeographic distribution of *Livistona* spp. [35] and *Adansonia gregorii* in Australia [36]. Hence our study includes the three alternative hypotheses mentioned above for assessing the human-assisted introduction of *A. farnesiana* in Australia prior to British colonization of the continent in 1788.

## 2. Material and methods

### 2.1. Sampling and area definition for biogeographic analysis

We obtained genetic data from microsatellites for 172 samples from 18 populations of *A. farnesiana* (table 1). These samples came from our collections throughout Australia, Spain, Madagascar and the Mascarene islands in the Indian Ocean region, India, Fiji and Mexico, and were supplemented with herbarium samples. We obtained South American and Eastern Atlantic samples from herbarium collections. We were unable to collect or obtain herbarium samples from continental Africa, the Arabian Peninsula and West Asia. Hence our samples do not comprehensively represent the plant's entire pan-tropical distribution.

**Table 1.** Collection localities of populations of *Acacia farnesiana* (L.) Willd. used in this study and groupings resulting from cluster analysis using Structure. Samples were collected from populations in northern Australia, South Central Mexico, Spain, Madagascar, Réunion, India and Fiji. Voucher specimens were retained for a representative subset of individuals and these were deposited in the National Herbarium of Victoria (MEL). These samples were supplemented with herbarium specimens from additional populations in the Americas, Cape Verde and the Philippines. For details of individual specimens and vouchers, see the electronic supplementary material, appendix S1.

population	locality	no. individuals	latitude	longitude	predominant cluster (*K* = 2)	predominant cluster (*K* = 5)
Arizona	University of Arizona, cultivated specimens	5	32.2327° N	110.954° W	B	3
	Sauceda Mountains	1	32.4979° N	112.599° W	A	unassigned
	Maricopa County	3	33.36° N	112.03° W	A	3
	Tortilla Flat	1	33.5° N	112.5° W	B	unassigned
Baja California	Sierra de la Gigante	1	25° N	110.95° W	B	unassigned
	Bahia Conception	1	26.8° N	111.88° W	B	2
	Cerro Colorado	1	27.30583° N	112.848° W	B	unassigned
	Sierra San Francisco	1	27.60667° N	113.027° W	B	2
	Unspecified	1	30.84063° N	115.28° W	B	2
	Unspecified	1	Approx. 26° N	Approx. 111.7° W	B	unassigned
Northwest Mexico	San Luis Potosi: Municipio Matehuala	1	23.71667° N	100.717° W	B	unassigned
	Coahuila: Rio Canon	1	26.98833° N	102.064° W	B	unassigned
	Sonora: El Aguajito− Rancho El Palmar	2	28.47° N	109.21° W	B	unassigned
	Sonora: Altar Municipio	1	31.46583° N	111.601° W	B	unassigned
	Michoacán: Numaran	1	20.25° N	101.94° W	unassigned	unassigned
	Hidalgo	1	20.7° N	99° W	unassigned	unassigned
	San Luis Potosi	1	22.2° N	101° W	B	unassigned
	Nuevo Leon	1	25.7° N	99.5° W	unassigned	unassigned
Veracruz	Veracruz: Los Negritos	10	18.83833° N	96.07° W	B	2
South Central Mexico	Oaxaca: Miahuatlan	1	16.30167° N	96.2856° W	B	unassigned
	Puebla: Atzitzihuacán− Morelos: Tlayecac	20	18.79° N	98.72° W	A	5
	Oaxaca	1	17.1° N	96.7° W	unassigned	unassigned
Central America	Costa Rica: Guanacaste: La Cruz	1	10.86° N	85.63° W	B	2
	Guatemala: Baja Verapaz− Chiquimula	2	14.97° N	89.92° W	B	unassigned
	Mexico: Campeche	1	18.22722° N	89.4533° W	A	3
Northern South America	Brazil: Maranhão: Alcântra	1	2.409° S	44.414° W	1	3
	Ecuador: Manabí: Jaramijó	1	0.12306° S	80.2186° W	1	3
	Guyana: Demerara: Mahaica region	1	6.633° N	57.917° W	1	3
Caribbean	Netherlands Antilles: Saba	1	17.626° N	63.249° W	2	unassigned
	Puerto Rico	2	18.22393° N	66.604° W	1	3
Southern South America	Paraguay: Central: Tavarory	1	25.472° S	57.551° W	2	unassigned
	Brazil: Mato Grosso do Sul: Campo Grande	1	20.443° S	54.646° W	1	3
	Brazil: Minas Gerais: Jaiba	1	15.333° S	46.833° W	2	2
	Brazil: Goias: Flores de Goias	1	14.449° S	47.05° W	2	2
	Bolivia: La Paz: Abel Iturralde	1	14.4167° S	67.05° W	2	2
	Brazil: Goias: Posse	2	14.067° S	46.487° W	2	2
	Brazil: Bahia: Tucano	1	10.9667° S	38.8° W	1	3
	Peru: Ucayali: Coronel Portillo	1	8.35° S	74.5667° W	1	3
Spain	Playa de las Palmeras−El Chuche	8	37.18° N	1.93° W	1	unassigned
	Crevillent−Via verde del Noroeste	9	38.11° N	0.99° W	1	unassigned
	Alzira	1	39.16° N	0.46° W	1	unassigned
Atlantic Islands	São Tomé e Príncipe: Praia Gamboa	1	0.334353° N	6.722458° E	1	3
	Cabo Verde: Santo Antão Tarrafal	1	16.6° N	24.2722° W	1	unassigned
Mascarene Islands	Réunion: Fluerimont	1	21° S	55° E	1	3
	Madagascar: Ambanja−Ankify Road	6	13.61° S	48.45° E	1	3
	Madagascar: Nosy Be	2	13.3833° S	48.2° E	1	3
	Madagascar: Diego Suarez−Ramena	7	12.32° S	49.35° E	1	3
India	India: Khatuali Canal	2	29.2504° N	77.1797° E	1	3
Philippines	Mt. Palay, Palay National Park and Bataan Province	2	14.7° N	120.6333° E	unassigned	5
Fiji	Natawarau	7	17.5131° S	177.5539° E	1	3
	Volivoli− Vunitogoloa	13	17.34° S	178.14° E	1	3
Northwestern Australia	Northern Territory: 130 km W of Alice Springs	3	23.6847° S	132.7094° E	2	1
	Western Australia: Mardie 2 Mile Mill	2	21.1911° S	116.0167° E	2	1
	Western Australia: 20 km from Whim Ck	3	20.8343° S	117.8442° E	2	1
	Northern Territory: 5 km E of ‘Soudan’	2	20.0295° S	137.0657° E	2	1
	Western Australia: 30 km SW Onslow	1	21.694° S	114.9184° E	2	1
	Western Australia: 12 km N of Sandfire Roadhouse	1	19.6623° S	121.0909° E	2	1
	Western Australia	1	19° S	123.5° E	2	1
	Western Australia: Halls Creek	2	18° S	128° E	2	1
	Western Australia: 5 km E of Fitzroy River town	1	18.2331° S	125.5876° E	2	1
	Western Australia: 180 km E of Halls Creek	1	17.944° S	128.8816° E	2	1
	Western Australia: Willare Roadhouse	1	17.7336° S	123.6484° E	2	1
	Northern Territory: Katherine	1	14.4625° S	132.2594° E	2	1
Central and Northeastern Australia	Queensland: 31 km W of Cloncurry	1	20.7584° S	140.2327° E	2	1
	Queensland: Koon Kool Station, Hughenden	3	20.674° S 7	144.3401° E	2	1
	Queensland: Charters Towers	2	20.0105° S	146.1665° E	2	1
	Northern Territory: 15 km NE Kalkarindji	3	17.4379° S	130.9372° E	2	1
	Northern Territory: 34 km E Top Springs	3	16.7607° S	131.6098° E	2	1
Southeastern Australia	NSW: Kirramingly Nature Reserve^a^	5	29.4622° S	149.8419° E	2	1

^a^Locality of seed collection. For locality details of cultivated voucher specimen, see electronic supplementary material, appendix S1.

We delineated the samples by three broad regions: Americas, Australia and Old World. We used the very broad category of Old World to include samples from all areas outside of the Americas and Australia. Hence, although not historically accurate, this category included not just Asia, Eastern Atlantic islands and Europe, but also the Indian Ocean and Pacific Ocean islands. The sampling strategy was designed to include populations across the putative native and introduced range of *A. farnesiana* as defined in table 1. Field-collected leaf material was placed in sealed plastic bags containing silica gel.

### 2.2. DNA isolation and microsatellite genotyping

We isolated plant DNA using the DNeasy Plant Mini Kit (QIAGEN), and analysed the microsatellite loci Af18, Af24, Af05, Af38, Af19, Af32, Af25, Af03, Af10, Af26, Af47, Af14 and Af46 [37]. We followed the method of James *et al*. [38] for amplification of the loci, with modifications for multiplex PCR reactions using the Type-It Microsatellite PCR Kit (QIAGEN). Amplification reactions contained a final concentration of 1x Type-It PCR Master Mix, 0.075 µM each forward primer appended to the 454A sequence tag, 0.25 µM each reverse primer, 0.1 µM/multiplexed locus of 454A sequencing tag labelled with 6-FAM, NED, HEX or PET (Applied Biosystems, Foster City, CA, USA). Thermal cycling followed the instructions provided with the Type-It Kit. Multiplexed amplicons were run by Macrogen Inc. (Seoul, Korea) or the Australian Genome Research Foundation (Melbourne, Australia) on a 3730XL sequencer (Applied Biosystems), with a GS500LIZ size standard. Peaks were scored using the Geneious microsatellite plugin version 1.0.0 (Biomatters Ltd, Auckland, New Zealand).

### 2.3. Genetic diversity

We used genalex v. 6.41 [39] to calculate genetic diversity parameters for populations, regions and the entire range. Genetic diversity parameters included the number of alleles per locus, effective number of alleles per locus, number of private alleles per locus, Shannon's information index, observed heterozygosity, expected heterozygosity under Hardy−Weinberg equilibrium and Wright's allelic fixation index (*F*: inbreeding coefficient).

### 2.4. Geographical structure

We used a Bayesian approach, implemented in Structure v. 2.3.3 [40] to determine the geographical structuring of genetic diversity within *A. farnesiana*, and to associate Old World and Australian samples with their American source populations. The likelihood of different values for the number of clusters (*K*) was calculated under an admixture model, with loci assumed to be unlinked. Multiple runs at the appropriate value(s) of *K* were combined using Clumpp v. 1.1.2 [41] and plotted graphically using Distruct v. 1.1 [42].

We used Analysis of Molecular Variance (AMOVA) [43] to determine the statistical significance of genetic structure at the regional level and at the population level. We used genalex v. 6.41 [39] to conduct this analysis. Populations with less than five individuals were excluded. Analyses were based on the *R*_ST_ measure of genetic diversity, with 9999 permutations.

### 2.5. Coalescent modelling

We used coalescent modelling to assess migration rates between geographically defined populations, effective population sizes and divergence times. Different programs model the coalescent process to estimate a subset of these parameters. We used migrate-n [44] to estimate rates of gene flow between populations and effective population sizes of these populations. We used IMa2 [45] to estimate divergence time between genetically distinct regions (determined from analyses of geographical structure outlined above), and to estimate gene flow rates and effective population sizes of these regions, and the ancestral population. Analyses were run on an SGI Altix XE Cluster through the Victorian Life Sciences Computing Initiative (VLSCI).

#### 2.5.1. Analysis with migrate-n

We used genotypes of individuals at each locus in a coalescent model to estimate migration rates between populations (*m* = **m/μ**, where **m** is the rate of migration for each gene copy and **μ** is the mutation rate per gene copy per generation), and population sizes for extant and ancestral populations (*θ* = 4**Nμ**, where **N** is the effective population size for a diploid species) [46,47].

Populations were pooled to reduce the number of parameters being estimated. Three pooled populations were considered in the Americas based on regional biogeography, rather than national boundaries: Arizona and Northwest Mexico (north of the Trans-Mexican Volcanic Belt); South Central Mexico (from the Trans-Mexican Volcanic Belt to the Isthmus of Tehuantepec); and Central and South America plus the Caribbean Islands (including Campeche, Mexico, which is south of the Isthmus of Tehuantepec). The other two pooled populations considered were the Old World, as defined above, and Australia. Estimated migration rates were considered to be significant if zero was not within the 95% highest posterior density (HPD) interval.

A simple stepwise mutation (SSM) model of molecular evolution was used for each of the microsatellite loci, and mutation rates were allowed to vary among loci. For each hypothesized divergence, we ran two parallel Bayesian MCMC analyses of four heated chains (1.0, 1.5, 3.0 and 1 000 000.0), with independent random starting points. Starting parameters were based on a UPGMA tree and *F*_ST_. Bayesian uniform priors for *θ* and *m* were bound between 0 and 50, and between 0 and 100, respectively. A burn-in of 50 000 steps was followed by a further 50 000 recorded steps with sampling every 100 steps for each locus. Convergence on stationary distributions of parameters was assessed based on the similarity of posterior distributions of independent runs, and the effective sample size.

#### 2.5.2. Analysis with IMa2

Divergence time between genetically distinct regions (determined from analyses of genetic structure, above), along with gene flow rates and effective population sizes, were estimated using IMa2 [45]. We used an SSM model of molecular evolution for each microsatellite locus. Four preliminary MCMC analyses with independent random starting points were run with 100 heated chains, under a geometric heating scheme with heating parameters ranging from 0.3 to 0.995, with 300 000 steps of burn-in, followed by one sampled genealogy, at which point the Markov chain state was saved to a file. Each of these Markov chain state files was then used as a starting point for five further runs under the same conditions for a total of 20 runs. For each of these runs, a further short burn-in of 10 000 steps was used, to ensure that each run had moved to an independent starting point before saving genealogies. Following this extra burn-in, each MCMC analysis continued for a further 5000 saved genealogies, saving every 100 steps, giving a total of 100 000 saved steps.

Coalescent parameters were estimated to be the peak of the posterior probability distribution from the combined MCMC runs, and the confidence interval was determined as the 95% HPD. Divergence models were assessed using likelihood ratio tests, with likelihood ratios expected to follow a *χ*^2^-distribution. Specifically, we tested for differences in effective population size of extant and ancestral populations, non-zero migration rates and asymmetrical migration rates. Relative divergence times, population sizes and rates of gene flow were converted into absolute values assuming a generation time of 3 years [48], and mutation rates of either 2.4 × 10^−4^ mutations/marker/generation (based on direct observation of the microsatellite mutation rate in wheat [49]) or 5.0 × 10^−4^ mutations/marker/generation (considered to be the average mutation rate over many species [50,51]), as there were no fossil data available at the species level to calibrate mutation rates.

## 3. Results

### 3.1. Genetic diversity

At the broad regional level, we found genetic diversity in *A. farnesiana* to be highest in the Americas, and lowest in the Old World (table 2). The number of alleles per locus and expected heterozygosity were both highest in the Americas. Private alleles were detected in the Americas and Australia, but not in the Old World. Within populations in the Old World, genetic diversity was slightly higher in Spain than in other populations. Populations in the Old World all had strongly negative values for *F*.

**Table 2.** Population statistics (mean ± s.d. across loci) for each population of *Acacia farnesiana* (L.) Willd., for each region, and for the entire species range. *n,* number of samples; *N*_a_, number of alleles per locus; *N*_e_, effective number of alleles per locus; *I*, Shannon's information index; *H*_o_, observed heterozygosity; *H*_e_, expected heterozygosity under Hardy–Weinberg equilibrium; *UH*_e_, unbiased expected heterozygosity = (2*n*/(2*n* − 1)) * *H*_e_; *F*, Wright's allelic fixation index.

region	population	*n*	*N_a_*	*N*_e_	private alleles	*I*	*H*_o_	*H*_e_	*UH*_e_	*F*
Americas	Arizona	9.84 ± 0.10	4.46 ± 0.56	2.69 ± 0.37	0.385 ± 0.870	1.06 ± 0.14	0.43 ± 0.11	0.54 ± 0.06	0.57 ± 0.06	0.26 ± 0.15
	Baja California	5.00 ± 0.00	2.85 ± 0.39	2.07 ± 0.28	0.077 ± 0.277	0.75 ± 0.13	0.42 ± 0.09	0.42 ± 0.07	0.47 ± 0.07	0.05 ± 0.13
	Northwest Mexico	10.31 ± 0.26	5.39 ± 0.45	3.53 ± 0.39	0.308 ± 0.751	1.36 ± 0.11	0.58 ± 0.06	0.67 ± 0.04	0.70 ± 0.04	0.11 ± 0.08
	Veracruz	9.92 ± 0.08	3.85 ± 0.34	2.67 ± 0.26	0.077 ± 0.277	1.05 ± 0.11	0.58 ± 0.09	0.57 ± 0.05	0.60 ± 0.05	−0.00 ± 0.14
	South Central Mexico	20.77 ± 0.12	3.46 ± 0.43	2.28 ± 0.34	0.154 ± 0.376	0.79 ± 0.16	0.50 ± 0.12	0.42 ± 0.08	0.43 ± 0.09	0.01 ± 0.17
	Central America	3.69 ± 0.18	3.15 ± 0.30	2.41 ± 0.24	0.231 ± 0.439	0.94 ± 0.10	0.53 ± 0.10	0.54 ± 0.05	0.62 ± 0.05	0.05 ± 0.13
	Northern South America	2.23 ± 0.23	1.54 ± 0.14	1.40 ± 0.12	0.000	0.32 ± 0.09	0.37 ± 0.12	0.22 ± 0.06	0.30 ± 0.09	−0.61 ± 0.11
	Caribbean	2.62 ± 0.14	2.23 ± 0.36	2.03 ± 0.31	0.308 ± 0.630	0.59 ± 0.17	0.41 ± 0.13	0.34 ± 0.09	0.42 ± 0.11	−0.20 ± 0.14
	Southern South America	7.00 ± 0.54	4.23 ± 0.60	3.12 ± 0.45	0.308 ± 0.855	1.13 ± 0.16	0.35 ± 0.08	0.58 ± 0.07	0.62 ± 0.07	0.39 ± 0.10
	total	71.39 ± 1.05	8.39 ± 0.61	3.30 ± 0.40	4.154 ± 1.864	1.40 ± 0.11	0.48 ± 0.08	0.64 ± 0.04	0.65 ± 0.04	0.27 ± 0.11
Old World	Spain	18.00 ± 0.00	1.92 ± 0.29	1.77 ± 0.25	0.000	0.48 ± 0.14	0.45 ± 0.14	0.30 ± 0.08	0.31 ± 0.09	−0.49 ± 0.19
	Atlantic Islands	1.54 ± 0.18	1.46 ± 0.22	1.41 ± 0.20	0.000	0.34 ± 0.11	0.42 ± 0.14	0.23 ± 0.07	0.33 ± 0.11	−0.82 ± 0.08
	Mascarene Islands	15.85 ± 0.10	1.77 ± 0.23	1.58 ± 0.18	0.000	0.40 ± 0.12	0.47 ± 0.14	0.26 ± 0.08	0.27 ± 0.08	−0.65 ± 0.17
	India	2.00 ± 0.00	1.46 ± 0.14	1.46 ± 0.14	0.000	0.32 ± 0.10	0.46 ± 0.14	0.23 ± 0.07	0.31 ± 0.10	−1.00 ± 0.00
	Philippines	1.39 ± 0.18	0.39 ± 0.18	1.32 ± 0.18	0.000	0.30 ± 0.09	0.39 ± 0.13	0.21 ± 0.08	0.33 ± 0.11	−0.78 ± 0.10
	Fiji	20.00 ± 0.00	1.62 ± 0.21	1.48 ± 0.15	0.000	0.34 ± 0.11	0.46 ± 0.14	0.24 ± 0.087	0.24 ± 0.08	−0.96 ± 0.02
	total	58.77 ± 0.38	2.62 ± 0.42	1.71 ± 0.21	0.000	0.51 ± 0.14	0.46 ± 0.14	0.30 ± 0.08	0.30 ± 0.08	−0.04 ± 0.10
Australia	Northwestern	19.15 ± 0.36	4.08 ± 0.66	2.65 ± 0.45	0.308 ± 0.598	0.94 ± 0.17	0.53 ± 0.12	0.49 ± 0.07	0.50 ± 0.08	0.07 ± 0.19
	Central and Northeastern	10.92 ± 0.08	3.23 ± 0.48	2.42 ± 0.29	0.000	0.89 ± 0.13	0.58 ± 0.13	0.51 ± 0.06	0.53 ± 0.07	−0.05 ± 0.21
	Southeastern	5.00 ± 0.00	2.00 ± 0.25	1.78 ± 0.18	0.000	0.54 ± 0.12	0.54 ± 0.14	0.35 ± 0.07	0.39 ± 0.08	−0.44 ± 0.20
	total	35.08 ± 0.37	4.46 ± 0.68	2.61 ± 0.39	0.538 ± 1.127	0.98 ± 0.15	0.55 ± 0.13	0.51 ± 0.07	0.52 ± 0.07	0.08 ± 0.20

### 3.2. Geographical structure

**Figure 1.**
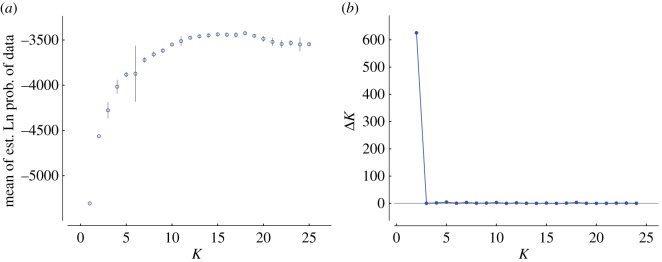
Comparison of Structure analysis results for genetic clustering of *Acacia farnesiana* (L.) Willd. for each value of *K* (number of genetic clusters) between one and 25. Simulations consisted of a burn-in of 50 000 iterations (an initial stage in the analysis where the data are not stored) followed by 100 000 Markov Chain Monte Carlo (MCMC) iterations (a measure of how long the analysis is run). Ten runs for each value of *K* were carried out on an SGI Altix XE Cluster through the Victorian Life Sciences Computing Initiative (VLSCI). (*a*) Mean and standard deviation of ln(likelihood) for each value of *K.* (*b*) Δ*K* [52] for each value of *K*, determined using Structure Harvester web v. 0.6.93 [55]*.*

**Figure 2.**
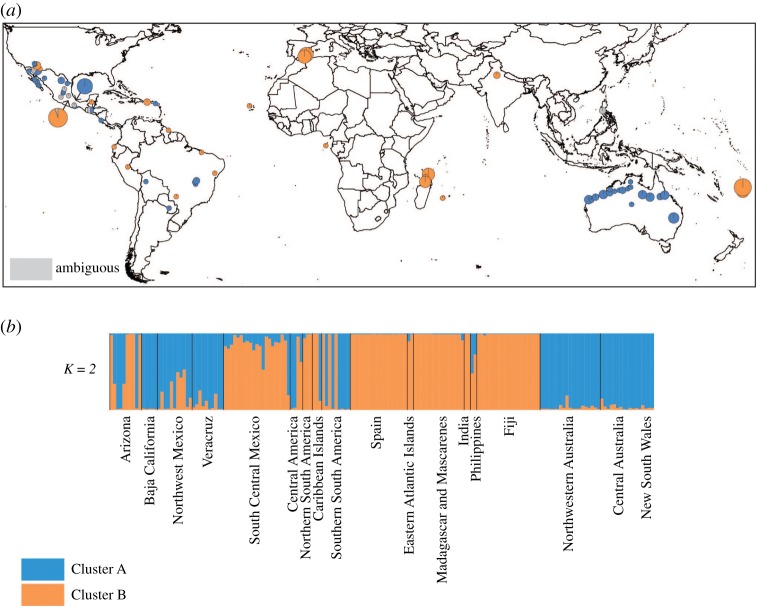
(*a*) Geographical centres of *Acacia farnesiana* (L.) Willd. populations, and the proportion of individuals within populations belonging to each genetic cluster, based on *K* *=* 2, visualized spatially using ArcGIS v. 10.0 (ESRI Inc.). Individuals are unassigned to clusters if their *Q*-value is less than 0.75, depicted in grey (*Q*-value is a score between 0 and 1 for each individual for inclusion in each cluster, adding up to 1 for each individual; for example, an individual with absolute certainty of belonging to a cluster would have a score of 1 for that cluster and 0 for all others). The size of the pie chart is proportional to the sample size. (*b*) Proportional assignment of *A. farnesiana* individuals to clusters based on Structure analysis with *K* *=* 2. Both clusters are broadly distributed in the Americas. Populations predominantly belonging to Cluster A are found in southern South America, Northwest Mexico, Baja California, Central America and Veracruz. Populations predominantly of Cluster B are found in northern South America, Puerto Rico and South Central Mexico (Puebla−Morelos). Outside of the Americas, Cluster A is found in Australia, while Cluster B is found in the Old World. Individuals from the Philippines are unable to be assigned to either cluster with a Q-value greater than 0.75.

Bayesian analysis using Structure inferred a maximum Δ*K* [52] at *K* = 2, indicating this to be the number of clusters that best explained the data. A peak at *K* *=* 2 in Structure analyses can be an artefact (e.g. [53,54]), so we also examined clustering based on the secondary peak at *K* = 5 (figure 1*a,b*). At *K* = 2, both clusters were broadly distributed in the Americas (figure 2). Outside of the Americas, Cluster A was found in Australia, while Cluster B was found in the Old World.

**Figure 3.**
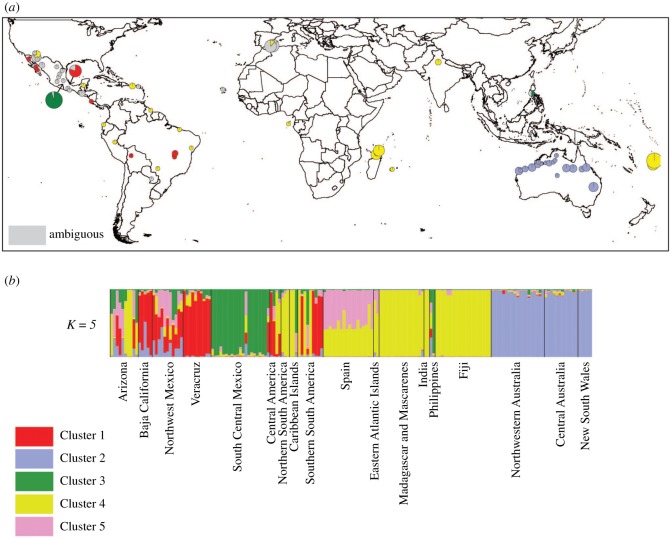
(*a*) Geographic centres of *Acacia farnesiana* (L.) Willd. populations, and proportion of individuals within populations belonging to each genetic cluster, based on *K* *=* 5, visualized spatially using PhyloGeo Viz [56] and ArcGIS v. 10.0 (ESRI Inc.). Individuals are unassigned to clusters if their *Q*-value is less than 0.75, depicted in grey (*Q*-value is a score between 0 and 1 for each individual for inclusion in each cluster, adding up to 1 for each individual; for example, an individual with absolute certainty of belonging to a cluster would have a score of 1 for that cluster and 0 for all others). The size of the pie chart is proportional to the sample size. (*b*) Proportional assignment of *A. farnesiana* individuals to clusters based on Structure analysis with *K* *=* 5. Clusters 1, 3 and 4 are all found in the Americas. Both Clusters 1 and 4 are widespread in the Americas, but with individuals from the same population typically assigned to the same cluster. Australian populations are assigned a unique cluster (Cluster 2). Cluster 3 is found in South Central Mexico. One individual from the Philippines has ambiguous ancestry; the other is assigned to Cluster 3. Individuals from Madagascar, Réunion, the east Atlantic, India and Fiji are assigned to Cluster 4. Individual plants from Spain and one plant from Cape Verde display approximately equal probability of assignment to Clusters 4 and 5. No samples can be assigned to Cluster 5 with a *Q*-value greater than 0.75.

At *K* = 5, Clusters 1, 3 and 4 were all found in the Americas (figure 3). Cluster 1 was only found in the Americas. Individuals from Madagascar, Réunion, the East Atlantic, India and Fiji were assigned to a single cluster (Cluster 4), suggesting a common source population. Individual plants from Spain and one plant from Cape Verde displayed approximately equal probability of assignment to Clusters 4 and 5 (figure 3*b*). One individual from the Philippines had ambiguous ancestry; the other was assigned to the same cluster as the population from South Central Mexico (Cluster 3). Australian populations were assigned a unique cluster (Cluster 2). No individuals from any population across the species distribution were assigned to Cluster 5 with a probability higher than 0.75.

A two-level AMOVA, with populations grouped into regions (Americas, Australia and Old World), partitioned 0% of total genetic variation among regions, 19% among populations, 75% among individuals and 6% within individuals. The variation between regions was not significant (*R_RT_* = −0.026; *p* = 1.0000). The variation between populations across the entire range, and between populations within regions was significant (*R*_ST_ = 0.172; *p* = 0.0001 and *R*_SR_ = 0.194; *p* = 0.0001). This means that although the populations we defined are genetically distinct, populations in different regions are no more genetically distinct than populations within the same regions.

### 3.3. Coalescent modelling

#### 3.3.1. Migration rate estimates migrate-n

**Figure 4.**
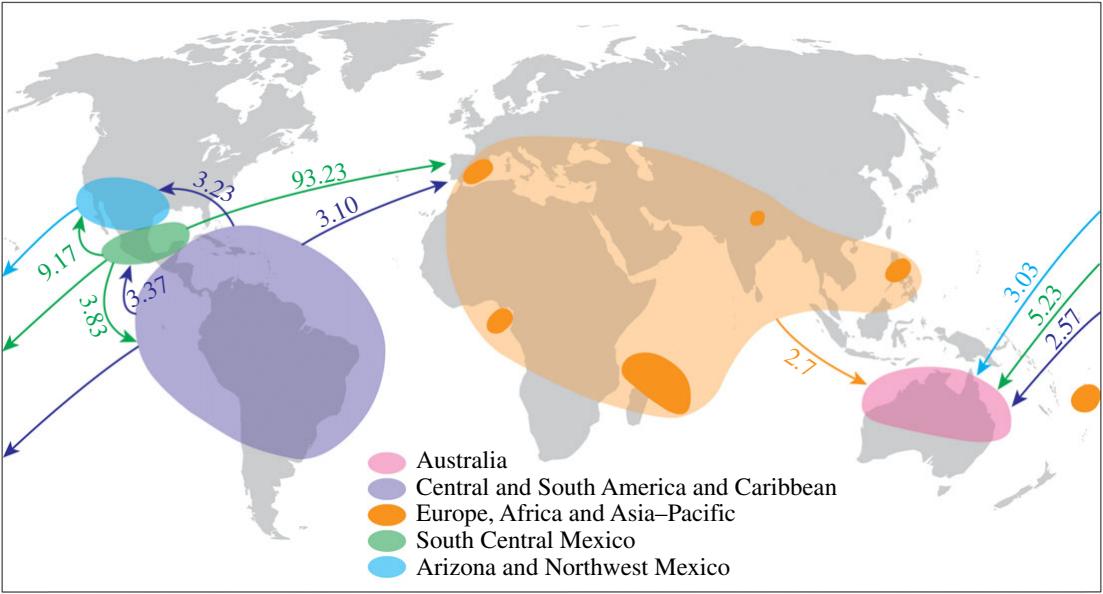
Migration rates (number of immigrants per generation) between populations of *Acacia farnesiana* (L.) Willd. as determined using migrate-n.

Using migrate-n, we detected significant, but low, levels of migration between regions within the Americas (table 3 and figure 4). Significant gene flow was detected from populations in the Americas to populations in the Old World and Australia (although this is inconsistent with the results from IMa2 analysis; see below). Based on the estimates of gene flow, we can infer the source populations for the introduced samples. The migration rate into the Old World from South Central Mexico was significantly higher than all other inferred migration rates. Rates of migration from each American region to Australia were moderate, but within this the migration rates from South Central Mexico, and from northwest Mexico and Baja California were marginally higher than the migration rates from Central and South America.

**Table 3.** Bayesian estimates (mode and 95% posterior probability interval) of migration rates (number of immigrants per generation) and mutation-scaled effective population size (*θ*) (a parameter that defines population size in terms of the diversity of genotypes) of *Acacia farnesiana* (L.) Willd. regional groups based on analysis with migrate-n for all loci combined. The migration direction is represented with the immigrant population on the columns.

	Arizona and Northwest Mexico	South Central Mexico	Central and South America	Australia	Old World	effective population size
Arizona and Northwest Mexico	—	2.23	2.30	3.03	1.77	2.32
		(0.33–4.07)	(0.33–4.13)	(1.00–5.00)	(0.07–3.47)	(1.17–3.53)
South Central Mexico	9.17	—	3.83	5.23	93.23	0.75
	(5.20–13.07)		(1.13–6.67)	(2.20–8.87)	(87.13–100.00)	(0.00–1.53)
Central and South America	3.23	3.37	—	2.57	3.10	1.85
	(0.93–5.60)	(0.40–5.80)		(0.67–4.33)	(1.07–5.07)	(0.13–5.86)
Australia	1.83	1.83	1.63	—	1.83	1.12
	(0.07–3.53)	(0.00–3.60)	(0.00–3.33)		(0.00–4.33)	(0.13–2.03)
Old World	2.70	3.17	1.83	2.70	—	0.42
	(0.53–4.87)	(1.00–5.33)	(0.00–3.60)	(0.67–4.60)		(0.00–1.27)

#### 3.3.2. Divergence time estimates from IMa2

Based on analyses of population genetic structure (see above), the only regions that had diverged sufficiently to allow calculation of divergence time were Australia and the Americas. Using IMa2, we estimated the divergence time between these regions as 795 (95% HPD confidence interval: 165–3795) or 1695 (360–8115) years ago, depending on the mutation rate used for scaling. The broad confidence intervals for divergence time make these results difficult to interpret (table 4). We tested for statistical significance of migration rates using likelihood ratio tests and found that they were not significant (tables 4 and 5). Although this is inconsistent with the non-zero migration rates inferred by migrate-n analysis between the American regions and Australia, it must be noted that the rates inferred by migrate-n were also low, and we did not run likelihood ratio tests to determine statistical significance in the migrate-n analysis.

**Table 4.** Population demographic parameters for the divergence between *Acacia farnesiana* (L.) Willd. populations in the Americas and Australia based on coalescent analyses in IMa2. For each parameter, the mode of the posterior probability density is presented, with 95% HPD (highest posterior probability density) confidence interval in brackets. Parameters have been converted into absolute values, using estimated mutation rates. This is in contrast to table 3, where mutation-scaled effective population sizes have not been converted into absolute values.

assumed mutation rate (mutations/ generation)	divergence time (years)	effective population size in Americas	effective population size in Australia	ancestral effective population size	immigration into Australia (individuals/ year)	immigration into Americas (individuals/ year)
5.0 × 10^−4^	795 (165–3795)	1850 (950–3350)	450 (150–1450)	5550 (3150–15 350)	0.65 (0–4.43)	0.0013 (0–1.60)
2.4 × 10^−4^	1695 (360–8115)	3950 (2030–7160)	960 (320–3100)	11 900 (6730–32 800)

**Table 5.** Likelihood ratio tests of models of migration and effective population size based on IMa2 analyses of *Acacia farnesiana* (L.) Willd. populations in the Americas and Australia. Different migration models are compared to the full model (a model allowing unrestricted migration in both directions and allowing all effective population sizes to vary). Where the model being tested is significantly less likely than the full model (*p* < 0.05), it is marked with an asterisk. When the test is statistically significant, this represents significant migration rates, significantly different migration rates or significantly different population sizes, depending on the model being tested.

migration model	log(*P*)	no. terms	degrees of freedom	2 log-likelihood ratio
full model	1.658	5	—	—
migration rates equal	1.658	4	1	0
no coalescent migration from Americas to Australia	1.658	4	1	0
no coalescent migration from Australia to Americas	1.658	4	1	0
no migration	1.658	3	2	0
population sizes of Americas and Australia equal*	−104.8	4	1	213*
population sizes of Americas and ancestral population equal	0.1048	4	1	3.106
population sizes of Australia and ancestral population equal*	−136.7	4	1	276.8*
all population sizes equal*	−223	3	2	449.3*

#### 3.3.3. Effective population size estimates from migrate-n and IMa2

Estimates of *θ* (mutation-scaled effective population size) from migrate-n analysis varied from 2.32 in Arizona and northwest Mexico to 0.42 in the Old World, but these values have large confidence intervals and most of these are overlapping (table 3). Within our IMa2 analysis, we conducted likelihood ratio tests to determine whether differences in effective population sizes were statistically significant. We found that effective population sizes were significantly smaller for Australia than for either the Americas or the ancestral population. This is consistent with a founder effect following introduction from a source population in the Americas.

## 4. Discussion

Our results are consistent with published records that *A. farnesiana* originates in the Americas [11–13]. This region has the highest genetic diversity (expected and observed heterozygosity) and the highest effective population size (based on coalescent analyses). All genetic clusters inferred by Structure at *K* = 2 and *K* = 5 are found in at least one sample from the Americas with a Q-score of more than 50%, and only a subset of these clusters are found outside the Americas. The majority of genetic clusters are widespread within the Americas, with many individuals displaying admixture between clusters. This implies high levels of dispersal within the region.

We discuss our hypotheses regarding the introduction of the plant to different Old World locations and to Australia below.

### 4.1. Introductions from the Americas to Southern Europe

The genetic data support our hypothesis that the introductions of *A. farnesiana* to southern Europe were via colonial interactions with the Americas. This is consistent with historical accounts of cultivation of the species in Italy and Spain as an ornamental during the seventeenth century [4,14]. The source populations for these introductions were probably from Central America, South America or the Caribbean Islands, based on assignment to similar genetic clusters in the Structure analysis. At a broader geographical scale, estimates of migration rates using migrate-n show a high probability of South Central Mexico as a source population for introductions to the Old World. This could be driven by the inclusion of samples from the Philippines with the Old World at this broad clustering level, and genetic similarities between samples from the Philippines and South Central Mexico (see below).

### 4.2. Secondary introductions via Southern Europe to other parts of the Old World

The genetic data support the hypothesis that the plant underwent secondary introductions to other parts of the Old World from southern Europe. Samples from Spain show the highest genetic diversity. Other populations from the Eastern Atlantic Islands, Madagascar and Mascarene Islands, and India are genetically similar to those from Spain and contain a subset of this diversity. This could be the result of an initial introduction to Spain, with an associated population bottleneck followed by subsequent spread to other populations and further bottlenecks. Alternatively, the higher genetic diversity in Spain could be the result of multiple introductions, with only one of these introductions involving other parts of the Old World. The high level of admixture of genetic clusters at *K* *=* 5 in Spain would be consistent with the latter scenario.

The dispersal of *A. farnesiana* to Asia from Spain may have followed sixteenth century Mediterranean trade routes connecting southern Europe with North Africa, Arabia and India [57]. The *A. farnesiana* populations in the Eastern Atlantic islands, Madagascar and Mascarene islands in the Indian Ocean, and Fiji in the Pacific Ocean are all part of the same genetic cluster that includes southern Europe and the Indian subcontinent. This corresponds with the expansion of Portuguese trade along Africa's Atlantic coast and in the Indian Ocean region during the sixteenth and seventeenth centuries, and followed subsequently by Dutch, French and British trade and colonization in Africa, Asia and the Pacific during the eighteenth and nineteenth centuries. The plant's introduction to Fiji is ascribed to the gardening efforts of foreign traders and European missionaries during the 1860s [58], who may have introduced it from southern Europe or southern Asia.

For Southeast Asia, the genetic data demonstrate that introductions of *A. farnesiana* to the Philippines were independent of the introduction via southern Europe. The Structure analysis at *K* = 5 assigned samples from the Philippines to the same genetic cluster as plants from South Central Mexico (Puebla−Morelos). This genetic connection with South Central Mexico appears to be limited to the Philippines, with no further dispersal of this genotype to other Pacific islands or Old World locations. The Spanish galleon trade across the Pacific Ocean from Mexico from the sixteenth to the nineteenth centuries involved the introduction of numerous plants to the Philippines and the Mariana Islands (Guam) [59–61], and *A. farnesiana* may have been introduced during this period by people travelling between these two places.

#### 4.2.1. *Acacia farnesiana* in Australia

There are no historical records for the introduction of *A. farnesiana* to Australia. Some of the earliest botanical explorations of northern Australia [62,63] note the species as being widespread at that time in some areas, which suggests its arrival prior to British settlement [64–66]. Hence, several Australian sources treat the plant as indigenous (e.g. [32,67]).

We tested three alternative hypotheses to explain the pre-British presence of *A. farnesiana* in the continent: (i) arrival via southeast Asia through colonial Portuguese or Spanish interactions; (ii) direct arrival from the Americas through European colonial voyages or (iii) pre-European arrival either through oceanic or human-assisted dispersal.

#### 4.2.2. Arrival via Southeast Asia

The combined genetic and historical evidence does not support the hypothesis of *A. farnesiana* introductions from Old World colonial networks in Southeast Asia. If the Australian populations had arrived through Spanish or Portuguese colonial trade networks, they would most probably share a genetic cluster with populations from the Old World or Southeast Asia. However, the genetic clustering in the Structure analysis at both *K* *=* 2 and *K* = 5 shows that the populations from Australia form a different genetic cluster from the Old World or the Philippines populations, hence suggesting a separate introduction.

#### 4.2.3. Direct arrival from the Americas through European colonial voyages

The combined genetic and historical evidence does not support the hypothesis of a direct introduction of *A. farnesiana* to Australia from the Americas through European colonial voyages or subsequent interactions. The divergence times based on the IMa2 coalescent analysis are difficult to interpret due to the large confidence intervals, but suggest introduction prior to European voyages and colonial interactions across the Pacific Ocean. The results of the Structure analyses also do not support direct arrival in Australia from the Americas through European colonial trade. The Australian population is not in the same genetic cluster as any of the other populations that are associated with European introduction.

#### 4.2.4. Pre-European arrival from the Americas

The genetic data offer strong evidence for a pre-European arrival from the Americas. This could have been through chance via oceanic dispersal, or through human agency. Genetic matching does not correspond with Spanish and Portuguese colonial activity in the Americas or in Southeast Asia, as discussed above. Estimates of migration rates between Australia and the Americas, using migrate-n and IMa2, are low or non-significant. At *K* = 5, Australian populations are assigned to Cluster 2. A small number of samples from northwest Mexico and Baja California have Q-values above 0.5 for assignment to this cluster, and these may reflect the source populations for earlier introduction to Australia. These results could imply that following an early dispersal event from northwest Mexico to Australia, the populations subsequently diverged due to isolation or due to no further introductions from the Americas.

### 4.3. The enigma of *Acacia farnesiana*'s arrival in Australia

How *A. farnesiana* arrived in Australia remains a historical enigma. It may be that a single chance event of oceanic dispersal [68] brought the plant's seeds from northwest Mexico across the Pacific Ocean to northern Australia, followed by gradual spread inland through wind, water, animals, birds and humans. Transoceanic dispersal by birds from Mesoamerica to Australia appears unlikely, since there are no records of bird migrations between these two regions [69]. Alternatively, *A. farnesiana* may have arrived through pre-European human-mediated introduction [70]. Our results are consistent with this. Although this may also seem improbable due to lack of any historical or archaeological evidence of pre-European human interactions between the Americas and Australia, a growing body of research using linguistic and genetic analysis indicates, for example, that sweet potato (*Ipomoea batatas*) was transferred by Austronesian sailors from the Americas into Oceania in pre-Columbian times [34,71]. As noted earlier, the genetic analysis of *A. farnesiana* samples from Fiji showed they were relatively recent introductions from southern Europe or India [58] and therefore not the source of early dispersals to Australia. Additional sampling and genetic analysis of *A. farnesiana* from intermediate islands in the north and south Pacific between the Americas and Australia may offer some clues.

One question that arises in relation to pre-European human-mediated introduction of *A. farnesiana* in Australia is whether the plant has long-standing recognition or use by indigenous groups. Our fieldwork in northwest Australia indicates that some Aboriginal languages in the Kimberley region such as Miriwoong have an indigenous name for *A. farnesiana* (*moorloomboo*), and that it is identified as a native plant typically growing on black soil country [72]. Further investigations of names and uses of *A. farnesiana* in other Indigenous languages of northern Australia combined with genetic analyses may provide insights into how the plant may have arrived and spread inland.

## 5. Conclusion

Our study is significant in providing the first genetic analysis of a plant introduction into continental Australia within a historical time frame of probably more than 750 years. While not conclusive, it also demonstrates the remarkable possibility of human-mediated dispersal of *A. farnesiana* from the Americas across the Pacific Ocean well before the arrival of Europeans to Australia. There are other plant species associated by their names and Aboriginal stories with pre-British colonial introductions to northern Australia from Southeast Asia and possibly further away from other parts of the Indian Ocean region. These include moringa (*Moringa olifera*), commonly referred to as Koepanger's tree (Kupang being the capital of West Timor) [73] and tamarind (*Tamarindus indica*) [74], which is often associated with the activities of Makassan trepangers (collectors of sea cucumber) and their trade with Aboriginal groups in northern Australia [75,76]. With increasing recognition of the long history of anthropogenic influence on vegetation change in the Australasian region [35,36], our study forges a new frontier for investigating ancient and precolonial interactions between Australia and the Pacific world and the Americas through integration of genetic analysis of plant species from these regions with available historical data.

The importance of recognizing the role of pre-European human-assisted plant dispersal goes beyond the Australia–Pacific region, and has broader implications for biogeographic studies of disjunct plant distributions around the world. Debates regarding disjunct plant distributions usually assume this is due to chance or transoceanic dispersal (e.g. [68]). The role of human-assisted dispersal is typically discussed in the context of European trade expansion and colonization of various world regions [77,78] but pre- and non-European human-mediated dispersal is rarely considered a possibility. In the absence of evidence, we see no reason to favour hypotheses of passive transoceanic dispersal as more parsimonious than explanations involving pre-European human interactions. There are other species in the genus *Acacia s.l.* with disjunct intercontinental distributions, such as *Acacia heterophylla* and *Acacia koa* [3], which are assumed to be the result of chance dispersal, but these should be reconsidered by including alternative hypotheses of human-assisted dispersal using multidisciplinary datasets including genetic, ecological, archaeological, historical, linguistic and social data.

There is a small, but growing, body of the literature using this interdisciplinary approach to investigate the ancient human history behind the current biogeographic distributions of various plant species [79–84]. Further research of this kind may not only solve the enigma of arrival of *A. farnesiana* to Australia, but also demand fundamental reconsideration of the pre-European history of indigenous interactions throughout the world.

## Supplementary Material

Appendix S1

## Supplementary Material

Appendix S2

## Data accessibility

The datasets supporting this study have been uploaded as part of the electronic supplementary material.
