# Supplementary material for: Chance long-distance or human-mediated dispersal? How Acacia s.l. farnesiana attained its pan-tropical distribution
Source: R Soc Open Sci. 2017 Apr 12;4(4):170105. doi: 10.1098/rsos.170105 (PMC5414274; doi:10.1098/rsos.170105)
Supplement: Appendix S1 [file rsos170105supp1.docx]

*Journal of Biogeography*

**SUPPORTING INFORMATION**

**Chance long-distance or human-mediated dispersal? How *Acacia s.l. farnesiana* attained its pan-tropical distribution**

Karen L. Bell, Haripriya Rangan, Manuel M. Fernandes, Christian A. Kull, Daniel J. Murphy

**Appendix S1:** Collection details of *Acacia farnesiana* samples used in this study and associated voucher specimens.

| Country | State/Territory/Province | Locality | Collector | Collection number | Voucher | Latitude | Longitude |
| --- | --- | --- | --- | --- | --- | --- | --- |
| USA | Arizona | Pima County; Tucson, University of Arizona, directly east of Economics (bldg. #23), cultivated | W. J. Liesenbein | 9 | ARIZ 377639 | 32.2327° N | 110.954° W |
| USA | Arizona | Pima County; Tucson, University of Arizona, directly east of Economics (bldg #23), cultivated | W. J. Liesenbein | 8 | ARIZ 377640 | 32.2327° N | 110.954° W |
| USA | Arizona | Pima County; Tucson, University of Arizona, directly east of Economics (bldg #23), cultivated | W. J. Liesenbein | 7 | ARIZ 377641 | 32.2327° N | 110.954° W |
| USA | Arizona | Pima County; Tucson, University of Arizona, directly east of Economics (bldg #23), cultivated | W. J. Liesenbein | 6 | ARIZ 377642 | 32.2327° N | 110.954° W |
| USA | Arizona | Pima County; University of Arizona campus, North-East of Physics and Atmospheric Sciences building, across the Santa Rita Avenue, cultivated. | J. Vanova | 176 | ARIZ 389682 | 32.2327° N | 110.954° W |
| USA | Arizona | Pima County; BLM; Sauceda Mountains. 3.2 km (map) northeast of Coffeepot Mountain. North of Charco. | P. Holm | 928 | ARIZ 361691 | 32.4979° N | 112.599° W |
| USA | Arizona | Maricopa County | D. Jenke | 666 | ASU 279693 | 33.2932° N | 112.428° W |
| USA | Arizona | Maricopa County; Gilbert; Riparian Preserve at Water Ranch; north end of Pond #6 along Willow Way | R. Gutierrez | 2305 | ASU 273906 | 33.3622° N | 111.738° W |
| USA | Arizona | Maricopa County; Tempe; Arizona State University Arboretum; Lot 59 | R. Gutierrez | 1340 | ASU 273859 | 33.4253° N | 111.931° W |
| USA | Arizona | Maricopa County | Miller et al. | 7719 | MO 4286110 | 33.5° N | 112.5° W |
| Mexico | Baja California | Sierra de La Giganta. Rancho La Banderita, Mesa de Humi. | J. L. Leon de la Luz | 10716 | ARIZ 385239 | 25° N | 110.95° W |
| Mexico | Baja California Sur | Mulegé Municipio; Bahia Conception; c. Km 118, E side of highway at base of hill is shallow wash. | D. Valov | 58 | ARIZ 389577 | 26.8° N | 111.88° W |
| Mexico | Baja California Sur | Mulegé Municipio; West base of Cerro Colorado, 6 km east of San Ignacio on MEX 1 | A. L. Reina G. | 371 | ARIZ 358841 | 27.30583° N | 112.848° W |
| Mexico | Baja California Sur | Sierra San Francisco, 1.4km NW of San Francisco, just NE of Cerro Los Sirios, shallow basin of dacite outcroppings, rock, and gravel, Prosopis glandulosa scrub; San Francisco Cetenal 1:50,000 topographic quadrangle | M. A. Baker | 15180 | ARIZ 368270 | 27.60667° N | 113.027° W |
| Mexico: | Baja California |  | M. A. Baker | 15180 | ASU 262307 | 30.84063° N | 115.28° W |
| Mexico | Baja California Sur |  | D. Seigler | 16151 | - | Approx. 26° N | Approx. 111.7° W |
| Mexico | San Luis Potosi | Mpio. Matehuala. Ejido La Cabra; 3 km del entronque de la carretera Matehuala-Cedral; frente al predio de la UASLP | H. Ramirez T. | 9 | ASU 244748 | 23.71667° N | 100.717° W |
| Mexico | Coahuila | Rio Canon; 2.1 miles north of Cuatro Cienegas northern limits; Canon de la Agua | D. J. Pinkava | 5455 | ASU 66767 | 26.98833° N | 102.064° W |
| Mexico | Sonora | El Aguajito on slopes of Mesa Chiquita, 17.3 km northwest of Yécora | A. L. Reina G. | 576 | ARIZ 371990 | 28.4561° N | 109.0208° W |
| Mexico | Sonora |  | Reina et al. | 97-106 | MO 4932975 | 28.4953° N | 109.3953° W |
| Mexico | Sonora | Altar Municipio; Near Rancho San Francisco, between Pozo Verde and Sasabe | A. L. Reina G. | 1299 | ARIZ 370043 | 31.46583° N | 111.601° W |
| Mexico | Michoacán | Numaran | D. Seigler | 16105 | - | 20.25° N | 101.94° W |
| Mexico | Hidalgo |  | D. Seigler | 15971 | - | 20.7° N | 99° W |
| Mexico | San Luis Potosi |  | D. Seigler | 15961 | - | 22.2° N | 101° W |
| Mexico | Nuevo Leon |  | D. Seigler | 15747 | - | 25.7° N | 99.5° W |
| Mexico | Veracruz | Los Negritos | R. Segura | MFS5255.1-10 (10 samples) | - | 18.83833° N | 96.07° W |
| Mexico | Oaxaca | Miahuatlan | Hunn | OAX-260 | MO 6178804 | 16.30167° N | 96.2856° W |
| Mexico | Morelos | Tlayecac | R. Segura | MFS5252.1-10 (10 samples | - | 18.7545° N | 98.8703° W |
| Mexico | Puebla | Atzitzihuacan | R. Segura | MFS5253.1-10 (10 samples) | - | 18.8247° N | 98.576° W |
| Mexico | Oaxaca |  | D. Seigler | 15995 | - | 17.1° N | 96.7° W |
| Costa Rica | Guanacaste | La Cruz | M. Grayum et al. | 11288 | MO 4788903 | 10.86° N | 85.63° W |
| Guatemala | Chiquimula | Chiquimula | Veliz | 14341 | MO 4851883 | 15.0892° N | 90.3225° W |
| Guatemala | Baja Verapaz | Salama | Vega.J.J. | 92 | MO 6217047 | 16.3017° N | 96.2856° W |
| Mexico | Campeche |  | D. Alvarez M. | 353 | MO 5608234 | 18.22722° N | 89.4533° W |
| Brazil | Maranhão | Acântara: Caminhos de Alcantara | A. M. V. de Carvalho | 2074 | NY 391134 | 2.409° S | 44.414° W |
| Ecuador | Manabí | Jaramijó | T. Delinks | 410 | MO 5555603 | 0.12306° S | 80.2186° W |
| Guyana | Demerara - Mahaica Region | Atlantic Coastline W of Mahaica R. between seawall and hospital | B. Hoffman et al. | 735 | NY 391140 | 6.633° N | 57.917° W |
| Netherlands Antilles | Saba | The Bottom | S. A. Mori | 26071b | NY 866196 | 17.626° N | 63.249° W |
| Puerto Rico |  |  | D. Seigler |  | - | 18.22393° N | 66.604° W |
| Puerto Rico |  |  | D. Seigler |  | - | 18.22393° N | 66.604° W |
| Paraguay | Central | Tavarory, Rio Paraguay | L. R. Landrum | 8757 | NY 391319 | 25.472° S | 57.551° W |
| Brazil | Mato Grosso do Sul | Campo Grande. Lagoinha, Compo Grande | W. A. Archer & Aug Gehrt | 125 | NY 917232 | 20.443° S | 54.646° W |
| Brazil | Goias | Formosa. Fazenda Santa Fe. Km 35 da rodovia Formosa/ Alvorada do Norte. | B. A. S. Pereira & D. Alvarenga | 2969 | NY 917920 | 15.333° S | 46.833° W |
| Brazil | Goias | Flores de Goias. Testemunha de mata e | B. A. S. Pereira | 2616 | NY 917944 | 14.449° S | 47.05° W |
| Bolivia | La Paz | Abel Iturralde | Bourdy | 1802 | MO 5197542 | 14.4167° S | 67.05° W |
| Brazil | Goias | Posse. Fazenda Sabonete, propritario Sr. Silvio dos Santos Lacerada | M. Aparecida da Silva | 4413 | NY 917921 | 14.067° S | 46.487° W |
| Brazil | Goias | Posse. Fazenda Sabonete, propritario Sr. Silvio dos Santos Lacerada | M. Aparecida da Silva | 4421 | NY 917922 |  |  |
| Brazil | Bahia | Tucano | de Carvalho & Hind | 3857 | MO 4230460 | 10.9667° S | 38.8° W |
| Peru | Ucayali | Coronel Portillo | J. G. Graham & J. Schunke V. | 461 | MO 5309198 | 8.35° S | 74.5667° W |
| Spain | Andalucía; Almería | El Chuche, near turn off from N340a, travelling towards Benahadux. El Chuche is some 1.5km from the turnoff. | H. Rangan | ESP288 | MEL 2370371 | 36.9059° N | 2.4484° W |
| Spain | Andalucía; Almería | El Chuche, near turn off from N340a, travelling towards Benahadux. El Chuche is some 1.5km from the turnoff. | H. Rangan | ESP287 | MEL 2370346 | 36.9059° N | 2.4484° W |
| Spain | Andalucía; Almería | Los Gallardos, on the bed of Rio Antas, going towards Bédar; name of locality, La Zimbra | H. Rangan | ESP286 | MEL 2370361 | 37.1712° N | 1.9431° W |
| Spain | Andalucía; Almería | Same location as ESP283 and ESP 284, growing underneath the bridge, close to the riverbank nearer Villarico side | H. Rangan | ESP285 | - | 37.2443° N | 1.7784° W |
| Spain | Andalucía; Almería | Same location as ESP283, on the riverbed of Rio Almanzora, some 10m away | H. Rangan | ESP284 | - | 37.2450° N | 1.7788° W |
| Spain | Andalucía; Almería | Near Villarico, on the riverbed of Rio Almanzora, near the bridge (Punta del Rio) across the river connecting Villarico to Palomares | H. Rangan | ESP283 | MEL 2370342 | 37.2450° N | 1.7788° W |
| Spain | Andalucía; Almería | Near Playa de las Palmeras, close to San Juan de Los Terrenos, roughly 100m from the sign showing turn off to Rancho Munuera | H. Rangan | ESP282 | - | 37.3710° N | 1.6161° W |
| Spain | Andalucía; Almería | Near Playa de las Palmeras, close to San Juan de Los Terrenos, roughly 100m from the sign | H. Rangan | ESP281 | MEL 2370376 | 37.3711° N | 1.6466 |
| Spain | Región de Murcia; Murcia | Via verde del Noroeste, near Mirador del Agridulce, behind University of Murcia; closest point of entry to trail at Ponto Cabezo del Aire | H. Rangan | ESP280 | - | 38.0208° N | 1.1877° W |
| Spain | Región de Murcia; Murcia | Via verde del Noroeste, near Mirador del Agridulce, behind University of Murcia; closest point of entry to trail at Ponto Cabezo del Aire | H. Rangan | ESP279 | MEL 2370383 | 38.0211° N | 1.1877° W |
| Spain | Comunitat Valenciana; Alicante | Between Orihuela and Santomera (Murcia) | H. Rangan | ESP278 | - | 38.0816° N | 0.9881° W |
| Spain | Comunitat Valenciana; Alicante | After Rincón de Bonanza, near Orihuela | H. Rangan | ESP277 | MEL 2370357 | 38.0842° N | 0.9767° W |
| Spain | Comunitat Valenciana; Alicante | Between San Carlos and Orihuela | H. Rangan | ESP276 | - | 38.1079° N | 0.9327° W |
| Spain | Comunitat Valenciana; Alicante | Between Albatera and Orihuela, near Virgen del Camino, before San Carlos | H. Rangan | ESP275 | - | 38.141° N | 0.9306° W |
| Spain | Comunitat Valenciana; Alicante | Between Albatera and Orihuela, near Virgen del Camino, before San Carlos | H. Rangan | ESP274 | MEL 2370337 | 38.141° N | 0.9306° W |
| Spain | Comunitat Valenciana; Alicante | Between Albatera and Orihuela, near Virgen del Camino, before San Carlos | H. Rangan | ESP273 | MEL 2370336 | 38.1415° N | 0.9302° W |
| Spain | Comunitat Valenciana; Alicante | Between Crevillent and Albatera | H. Rangan | ESP272 | MEL 2370362 | 38.2138° N | 0.8354° W |
| Spain | Comunitat Valenciana; Valencia | Alzira | H. Rangan | ESP271 | MEL 2370360 | 39.1608° N | 0.4554° W |
| San Tome e Principe |  | Praia Gamboa |  |  | COI 4984 | 0.334353° N | 6.722458° E |
| Cabo Verde |  | Sando Antao Tarrafal |  |  | COI 9353 | 16.6° N | 24.2722° W |
| Réunion |  | Fluerimont | V. Rahajanirina | RUNF025 | - | 21° S | 55° E |
| Madagascar | Antsiranana | Ambanja | V. Rahajanirina | BANF017.i-v (5 samples) | - | 13.6167° S | 48.4667° E |
| Madagascar | Antsiranana | Diana: PK 10 Ambanja-Ankify road | V. Rahajanirina | BANF019.ii | MEL 2370368 | 13.6° S | 48.3833° E |
| Madagascar |  | Nosy Be | V. Rahajanirina | NOSF012.i-ii (2 samples) | - | 13.3833° S | 48.2° E |
| Madagascar | Antsiranana | Diana: Montagne des Français | V. Rahajanirina | DIEF008.i | - | 12.3° S | 49.333° E |
| Madagascar | Antsiranana | Diana: Montagne des Français | V. Rahajanirina | DIEF009.i | MEL 2370384 | 12.3° S | 49.333° E |
| Madagascar | Antsiranana | Diana: Montagne des Français | V. Rahajanirina | DIEF009.ii | - | 12.3° S | 49.333° E |
| Madagascar | Antsiranana | Diana: Baie des Sakalaves | V. Rahajanirina | DIEF011.i | MEL 2370377 | 12.267° S | 49.383° E |
| Madagascar | Antsiranana | Diana: Baie des Sakalaves | V. Rahajanirina | DIEF011.ii | - |  |  |
| Madagascar | Antsiranana | Diana: south of Diego Suarez | V. Rahajanirina | DIEF013.i | MEL 2370354 | 12.417° S | 49.35° E |
| Madagascar | Antsiranana | Diana: south of Diego Suarez | V. Rahajanirina | DIEF013.ii | - |  |  |
| India |  | Khatuali Canal | P. Krishen | 01301.2-3 (2 samples) | - | 29.2504° N | 77.1797° E |
| Philippines |  | Luzon Cavite Malabinti, Mt. Palay-palay National Park Ternate | Reynoso et al. | 17168 | K 000295883 | 14.7° N | 120.6333° E |
| Philippines | Bataan Province |  | Reynoso et al. | 3969 | K 000661561 | 14.7° N | 120.6333° E |
| Fiji | Viti Levu | Natawarau roadside stand | C. Kull | 010.i | MEL 2370339 | 17.5131° S | 177.5539° E |
| Fiji | Viti Levu | Natawarau roadside stand | C. Kull | 010.ii-vii (6 samples) | - | 17.5131° S | 177.5539° E |
| Fiji | Viti Levu | Vunitogoloa village | C. Kull | 009.i | MEL 2370331 | 17.3689° S | 178.0606° E |
| Fiji | Viti Levu | Vunitogoloa village | C. Kull | 009.ii-v (4 samples) | - | 17.3689° S | 178.0606° E |
| Fiji | Viti Levu | King’s Highway; 300m west of intersection with Volivoli loop road | C. Kull | 001 | MEL 2370338 | 17.3403° S | 178.1833° E |
| Fiji | Viti Levu | ~1km down Volivoli loop road (west side) | C. Kull | 002 | - | 17.3339° S | 178.1833° E |
| Fiji | Viti Levu | ~2km down Volivoli loop road (west side) | C. Kull | 003 | MEL 2370403 | 17.3303° S | 178.1797° E |
| Fiji | Viti Levu |  | C. Kull | 004 | - | 17.3303° S | 178.1797° E |
| Fiji | Viti Levu | At beginning of spur road to Volivoli Point; steep climb to hilltop | C. Kull | 005 | - | 17.3194° S | 178.1831° E |
| Fiji | Viti Levu | At beginning of spur road to Volivoli Point; steep climb to hilltop | C. Kull | 006 | - | 17.3194° S | 178.1831° E |
| Fiji | Viti Levu | where loop road crosses over to eastern shore. | C. Kull | 007 | - | 17.3175° S | 178.1831° E |
| Fiji | Viti Levu | East side of Volivoli loop | C. Kull | 008 | - | 17.32° S | 178.19° E |
| Australia | Northern Territory | Ormiston Gorge turn-off from Namatjira Drive (ca 130km W of Alice Springs) | R. van Klinken | NT4.1-3 (3 samples) | - | 23.6847° S | 132.7094° E |
| Australia | Western Australia | Mardie 2 Mile Mill | R. van Klinken | WA13.1-2 (2 samples) | - | 21.1911° S | 116.0167° E |
| Australia | Western Australia | 20km from Whim Ck | R. van Klinken | WA12.1-3 (3 samples) | - | 20.8343° S | 117.8442° E |
| Australia | Northern Territory | 5km East of "Soudan"; Barkly Highway | N. March | NT1.1-2 (2 samples | - | 20.0295° S | 137.0657° E |
| Australia | Western Australia | Ashburton River 30km SW Onslow | N. March | WA2.3 | - | 21.694° S | 114.9184° E |
| Australia | Western Australia | 12km N of Sandfire Roadhouse; only plant; roadside; bluebush plains | R. van Klinken | WA7.1 | - | 19.6623° S | 121.0909° E |
| Australia | Western Australia |  | R. van Klinken | WA8.1 | - | 19° S | 123.5° E |
| Australia | Western Australia | 20km marker west of Halls Ck | R. van Klinken | WA4.1 | - | 18.3411° S | 127.527° E |
| Australia | Western Australia | Ord River Crossing; before Halls Ck | R. van Klinken | WA5.1 | - | 18.223° S | 127.6669° E |
| Australia | Western Australia | 2 Mile Creek Crossing; 5km E of Fitzroy River town | R. van Klinken | WA6.1 | - | 18.2331° S | 125.5876° E |
| Australia | Western Australia | Duncan Road; 180km E of Halls Creek | N. March | WA1.1 | - | 17.944° S | 128.8816° E |
| Australia | Western Australia | Fitzroy R crossing; 1km Broome side of Willare Roadhouse | R. van Klinken | WA3.1 | - | 17.7336° S | 123.6484° E |
| Australia | Northern Territory | Katherine | K. Bell | 128 | MEL | 14.4625° S | 132.2594° E |
| Australia | Queensland | Barkly Highway; 31km W of Cloncurry | N. March | QLD2.2 | - | 20.7584° S | 140.2327° E |
| Australia | Queensland | Koon Kool Station, Hughenden | R. van Klinken | QLD3.1-3 (3 samples) | - | 20.674° S 7 | 144.3401° E |
| Australia | Queensland | Charters Towers | R. van Klinken | QLD4.1-2 (2 samples) | - | 20.0105° S | 146.1665° E |
| Australia | Northern Territory | Buntine Road; 15km NE Kalkarindji | N. March | NT3.1-3 (3 samples) | - | 17.4379° S | 130.9372° E |
| Australia | Northern Territory | 34km E Top Springs on Armstrong River | N. March | NT2.1-3 (3 samples) | - | 16.7607° S | 131.6098° E |
| Australia | Victoria | Royal Botanic Gardens Melbourne, cultivated | K. Bell | 134 | MEL 2370402 | 37.8319° S | 144.9858° E |
| Australia | Victoria | Royal Botanic Gardens Melbourne, cultivated | K. Bell | 135-138 (4 samples) | - | 37.8319° S | 144.9858° E |
